# Beri-Beri

**Published:** 1904-11-26

**Authors:** Louis Vintras

**Affiliations:** Physician to the French Hospital


### Hospital Clinics.

### BERI-BERI.

By Louis Vintras, M.D., B.Sc., Physician to the French Hospital.

Having in the course of several voyages to Brazil and the Guianas, had special facilities for studying this disease, which, through the importation of Chinese labour into South Africa, has of late come prominently before the English public, the following remarks may prove of interest at the present moment.

Beri-beri is a disease of the motor nerves beginning at the periphery. It is of the nature of an acute degeneration of the nerve tissue, causing a failure on the part of the nerve-endings to carry out their work properly. Thus the earliest and, throughout, most striking symptom of the disease is muscular inefficiency, ending in protracted cases in atrophy and subsequent hardening of the substance of the muscles, causing contractions and deformities of the limbs or, where the muscles of the chest become involved, causing death.

The gait of the patients is typical; it is uncertain and jerky, with a tendency to run. The appearance of the face is also striking. There is puffiness, a sallow complexion, and a vague look of fear in the eyes. In acute cases there is a tendency to general dropsy, effusions into serous cavities, and hæmorrhages from the alimentary canal. Recovery in very acute cases is rare, and in the very protracted cases doubtful. The great majority of cases, however, are not so severe, and when taken in time are easily cured. I have seen at one visit in the General Hospital at Paramaïbo (Dutch Guiana) 30 cases, all of which were recovering.

Beri-beri is so constantly present in certain parts that it has been considered endemic, and at times attacks such large numbers that it has been looked upon as epidemic. But it is neither endemic nor epidemic in the true sense of the words. It is primarily due to privations and faulty nutrition, and it will appear wherever individuals are the victims of such conditions, and will only attack those individuals. Until recently I accepted the current statement that beri-beri was imported into Dutch Guiana with the coolies brought over from the Dutch possessions in the East; but a closer knowledge of the special characteristics of the disease has led me to reject that theory. It was not the disease itself which the coolies from Java and Sumatra brought with them, but it was their faulty dietetic habits, the bad quality and insufficiency of their food, which, under the influences of hard work, unsanitary surroundings, and of a damp and depressing climate, made them as liable to the disease in Guiana as they had been in the East or would have been in any other part of the world under similar conditions. A striking illustration of how the disease makes its appearance was given me in the hospital at Paramaïbo. Among the patients suffering from beri-beri, two were escaped convicts from the French penal settlement at Cayenne. It takes a man over 20 days to make his way through the dense bush and over swamps, from the territory of the French colony to Paramaïbo. The escaped convicts depend during this time on roots and such like precarious food for their sustenance. Most of them die in their desperate attempt for freedom, a few manage to reach the Dutch settlement in an exhausted condition and are invariably found to be suffering from beri-beri. Again, in northern Brazil, where the population is extremely poor, the disease is very prevalent, while in the southern provinces of Brazil, which are much more prosperous, it is much rarer.

The frequent appearance of beri-beri among the crews of Norwegian vessels is well known and easily explained. As I have pointed out elsewhere, the laxity of the shipping regulations in Sweden and Norway is an incentive to undermanning, underfeeding, and the evasion of sanitary measures. So true is this that shipowners in other countries, and even in England, take on board their ships a few Scandinavian subjects, register their ships in Norway, and sail them under the Norwegian flag, thus materially lessening their expenses by evading the justly more stringent regulations of their own countries. This is the cause which makes beri-beri more frequent among Norwegian crews than on board the ships of any other European nation, and it, moreover, emphasises the fact that the disease will spontaneously make its appearance among men whenever the necessary conditions for its development exist.

It is thus seen that beri-beri is not an infectious disease, and that both white and coloured populations are equally liable to it. Moreover, as Professor Uchermann, the President of the Norwegian Commission on this disease has recently established, there is no essential difference between Asiatic beri-beri and ship beri-beri, as is held to be the case by many authorities. The difference in the symptoms on which it has been sought to lay so much stress, is a difference due simply to the conditions under which the patients find themselves, and not to any fundamental pathological difference. For the symptoms differ as largely among Asiatics as they do among the members of white crews, nor can it be said that the form it assumes is more severe with the one class of patients than with the other.

How varied in its course and general manifestations is the disease, the following instances will illustrate.

In November 1894, 20 Annamites were brought over from Antwerp to give performances in London. The venture was not successful and eventually they were found herding together in a slum in Westminster. Their food had consisted of a sack of rice a week and twice a week six pounds of meat divided among them. One of them had died of beri-beri and two others were suffering from the disease. Through the action of the French Consul, to whom the facts were reported, the troupe was sent over to France and from there back to Annam. One of the patients was too ill to leave and was admitted to the French Hospital. I reported the case at length at the time. He had evidently been suffering from the disease for some considerable period; both the lower limbs were paralysed and the movements of his hands impaired; the hardening and retraction of the muscles had set in and he was in a very precarious condition generally. He, however, survived and is still alive; his lower limbs are quite deformed and useless, the feet being curved inwards, his hands also are partially paralysed, but at this point the progress of the disease was arrested, his general condition improved, he put on flesh, and beyond his helpless condition he has for years shown no further signs of the disease.

In 1899 while I was at Pernambuco, a training ship of the Brazilian navy was there, among the crew of which beri-beri had broken out. The disease had made its appearance while the ship was stationed at Para, over 200 cases having occurred. As nothing seemed to stop its spread, the vessel had been ordered south to Pernambuco and the outbreak was then rapidly subsiding. The form of the disease was not severe and so far as I could ascertain there had been no deaths.

On June 11th, 1900, the barque *Fernbank*, bound for Glasgow from New Caledonia, put into Barbadoes with beri-beri on board. Nearly all the crew were stricken. The captain had died five days previously. The ship was 110 days out, and the crew had been early put on low rations. The disease had made its appearance about a month after leaving New Caledonia, when the cook had been taken ill. He had died a month later. Several of the crew were admitted to the Barbadoes Hospital; the remainder, some 10 in number, were sent home on the mail steamer, to which I was then medical officer, and were placed under my care. Most of the cases were slight, two more severe. However, 10 days later, when we reached England, all had greatly improved and were progressing rapidly towards recovery.

The first of these cases illustrates an important pathological point in connection with the disease, which is that when the damage done to the nerve endings has reached a certain point, though the progress of the disease may be arrested, the affected parts of the nerves do not recover, and that the paralysis and subsequent deformities are irremediable. The cases on the Brazilian training ship are curious, as the crew were near land, and indeed mostly in port, being supplied with food from the shore. But the supplies were probably made by contract, without proper supervision on the part of the authorities, and one must arrive at the conclusion that the food was of inferior quality or considerably tainted, since when the ship was ordered to Pernambuco, which allowed of different catering, the outbreak subsided. The fact that only one of the several factors that go to produce the disease was here present explains why, though a large number of men were affected, the cases were not severe. The third instance, that of the *Fernbank*, furnishes a very good example of ship beri-beri, and shows how severe and slight cases occur side by side, without any reason beyond the differences of the patients' constitutions.

This brings me to the causation of beri-beri. In all cases its origin may be traced to insufficient and unsuitable food in conjunction with lowering physical conditions. Where life is dependent for any length of time on preserved foods, preserved vegetables of the dry variety, and especially rice, and there are also present insanitary surroundings and depressing influences, the disease is likely to appear and to spread. An almost exclusively rice diet, especially where the rice has been kept long, is a great factor in the causation of beri-beri. I believe that rice, especially when in contact with the air, does not keep so long as we are apt to think, and that, without showing signs of deterioration recognisable to the naked eye, it undergoes a species of fermentation which causes it to lose much of its nutritive value. Wherever communities are largely dependent on rice for their nourishment, especially imported rice, the Government cannot exercise too stringent a supervision over this article of diet. But I do not think that it can be said that there is any particular food that is responsible for the causation of this disease. Beri-beri will appear wherever life is dependent for any length of time on foods, whether animal or vegetable, whose nutritive value has become impaired, more especially when people are at the same time subjected to heavy physical strain or to long exposure in debilitating climates. Our present knowledge of dietetics is too imperfect for us to formulate the exact relations between the different constituents of our foods and the different tissues of the body. Otherwise, knowing that it is the nerves which are primarily affected in beri-beri, we should be able to say what is the particular impairment in food generally which is responsible for the causation of this disease. This is the problem which has still to be solved.

